# Klippel-Trenaunay Syndrome Associated With Urinary Tract Hemangiomas: A Case Report

**DOI:** 10.7759/cureus.42797

**Published:** 2023-08-01

**Authors:** Mohammed Ramdani, Anouar El Moudane, Youness Tahri, Ahmed Jdaini, Achraf Benamou, Mohamed Mokhtari, Ali Barki

**Affiliations:** 1 Urology, Mohammed VI University Hospital Oujda, Oujda, MAR; 2 Urology, Faculty of Medicine and Pharmacy of Oujda, Oujda, MAR

**Keywords:** congenital, case report, recurrent hematuria, bladder hemangioma, klippel trenauney syndrome

## Abstract

The association between Klippel-Trenauney syndrome (KTS) and bladder hemangiomas is rare. The most common clinical manifestation is hematuria. The diagnosis is made from the characteristic cystoscopic appearance of the tumor. We report the case of a patient presenting recurrent macroscopic hematuria in the context of KTS. A cystoscopic evaluation revealed bladder hemangiomas. A conservative approach consisting of bladder irrigation and close follow-up was chosen as therapy. Conservative treatment of bladder irrigation and close follow-up is the recommended initial treatment of moderate and infrequent episodes of hematuria in this context. The more invasive therapeutic options have to be considered especially for frequent or life-threatening episodes of hematuria. This case suggests that conservative treatment may be effective in treating moderate and infrequent episodes of hematuria due to bladder hemangioma in the context of KTS. Further studies are required to adequately establish the effectiveness, limitations, and complications of each approach.

## Introduction

Klippel-Trenaunay syndrome (KTS) is a rare congenital disorder defined as a syndrome with capillary and venous malformations as well as limb overgrowth. Bladder hemangioma is a benign vascular tumor, probably arising from embryonic remnants of angioblastic cells [1]. The association of KTS and bladder hemangiomas is rare and has only been described in 3-6% of cases [2]. Its most frequent clinical manifestation is hematuria, and the diagnosis is usually based on the characteristic cystoscopic appearance of the tumor. Different treatments have been proposed; partial cystectomy used to be the standard therapy [3,4], and laser photocoagulation is also a feasible option for smaller lesions [5]. We report the case of a 26-year-old female who was admitted with recurrent macroscopic hematuria due to bladder hemangioma associated with KTS.

## Case presentation

A 26-year-old woman with KTS of the right lower limb was admitted to the hospital with recurrent macroscopic hematuria. The history of the disease includes numerous hospitalizations for acute anemia requiring repeated transfusions since the age of 20 years. The patient had no history of allergy or drug use and any family history of KTS.

**Figure 1:**
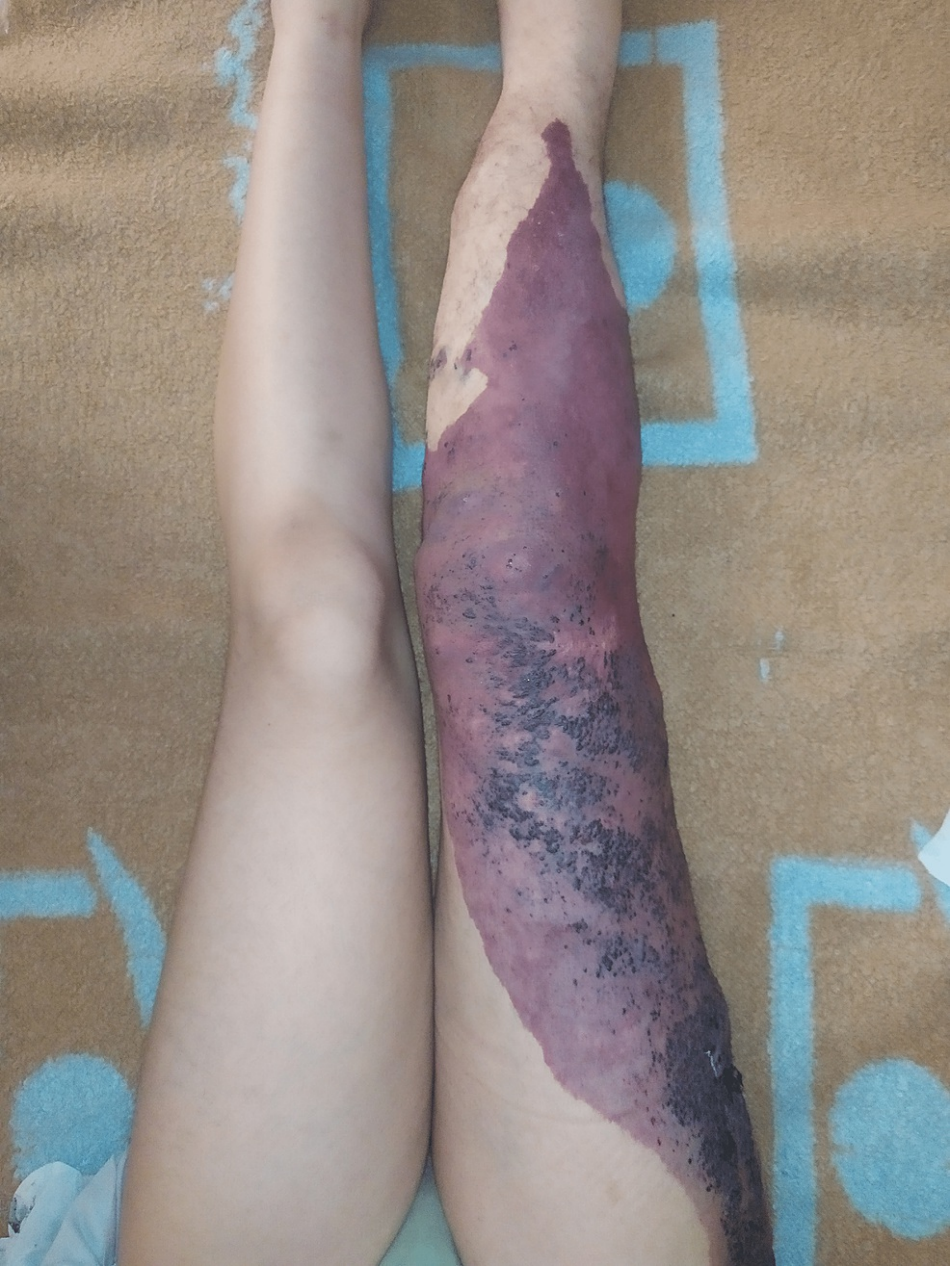
Cutaneous hyperpigmentation with raised margins (port wine stains) over the lateral aspect of the right lower limb, associated with dilated veins

Physical examination revealed a purple plaque with raised margins (port wine stains) over the lateral aspect of the right lower limb, associated with dilated veins over the outer side of the right lower limb since birth. On measurement, the right limb was 85 cm from the greater trochanter to the lateral malleolus, while the left limb was 82 cm (Figure 1).

Color Doppler of the lower right limb revealed an ectatic right great saphenous vein measuring 7.7 mm in diameter and individualization of multiple vascular structures in the right lower limb with venous flow. The angioscan of the lower right limb revealed multiple tortuous and ectatic vascular structures communicating with each other in the subcutaneous and with the superficial and deep subcutaneous soft tissues of the right lower limb reaching the homolateral gluteal and perineal region, creating true varicose veins, with no arterial communication, in relation to a venous malformation. The thoraco-abdomino-pelvic angioscan revealed thickening of the bladder wall as well as the spleen, kidneys, adrenal glands, liver, subcutaneous soft tissue, muscles of the lumbar wall, the gluteal, and perineal region.

**Figure 2:**
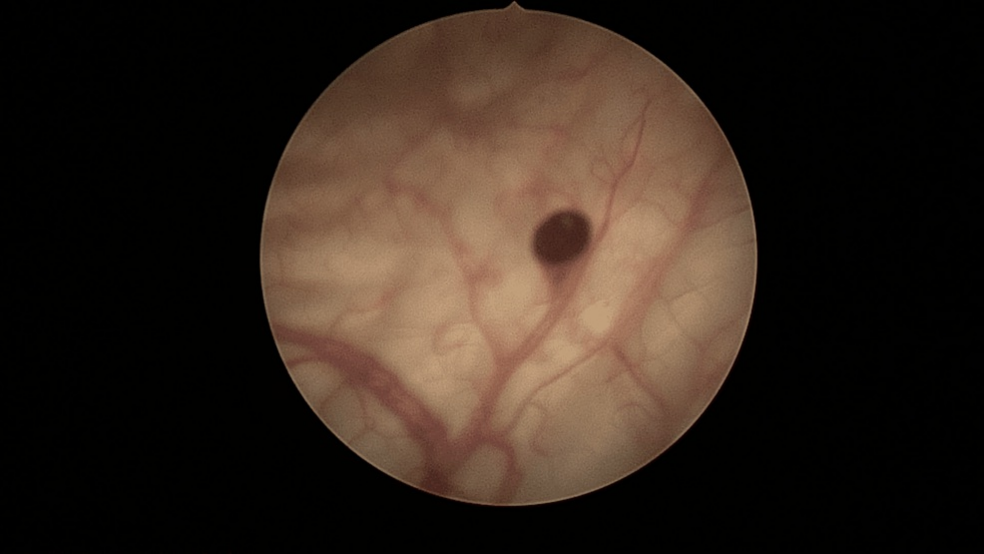
Endoscopic view of bladder angioma

On the urological side, the patient underwent a cystoscopy in the operating room of our urology department. She received spinal anesthesia, then she was positioned supine on the table with both lower limbs suspended down from the foot end of the table. A cystoscope was introduced into the bladder, and the diagnosis of bladder hemangiomas was made from the characteristic endoscopic appearance of the lesion; multiple circular buds less than 1 cm in diameter, brownish in appearance, located on the left lateral surface, and the bladder dome (Figure 2).

The patient was satisfied with the intervention. The postoperative evolution was without any particularity, and the patient was discharged from the hospital one day after the operation.

She was treated conservatively with elastic compression stockings. She was advised to keep the limb elevated when possible. For bladder hemangiomas, upon multidisciplinary counseling, we opted for a conservative approach consisting of bladder irrigation and close follow-up every 3 months.

## Discussion

Bladder hemangioma is a rare benign tumor [1]. The association of KTS and bladder hemangiomas has been described in 3-6% of cases [2]. The most common complaint is recurrent gross hematuria. The diagnosis is generally made from the cystoscopic appearance of the tumor [2,6,7].

A conservative approach of bladder irrigation and close follow-up was chosen as therapy, which is the recommended initial treatment for moderate and infrequent episodes of hematuria. Antifibrinolytic agents such as epsilon amino-caproic or tranexamic acid can also be used [5,8,9]. When conservative management is insufficient, a more invasive approach is considered especially for frequent or life-threatening hematuria.

Partial cystectomy used to be the standard therapy but carries significant morbidity [3,4]. Less invasive treatment options with their specific limitations and complications have been proposed, such as laser photocoagulation, which is a feasible option for smaller lesions, with some good reported results [5,10-12]. Transurethral resection and endoscopic biopsy have been strongly discouraged because of the risk of excessive bleeding [9,10,13,14]. Selective embolization of the internal iliac arteries is also an option, but recurrence due to the rapid development of collateral circulation and bladder or prostate infarction has been described [9,10,15]. Radiotherapy has only temporary results with significant morbidity [16].

## Conclusions

This case suggests that conservative treatment may be effective in treating moderate and infrequent episodes of hematuria associated with bladder hemangioma in the context of Klippel-Trenaunay syndrome but requires a close follow-up. The more invasive therapeutic options have to be considered especially for frequent or life-threatening hematuria.

However, further studies are required to adequately establish the effectiveness, limitations, and complications of each approach.
